# Oroantral fistula and genian mucosal flap: a review of 25 cases

**DOI:** 10.1016/S1808-8694(15)30756-4

**Published:** 2015-10-19

**Authors:** Roberto Campos Meirelles, Roberto Machado Neves-Pinto

**Affiliations:** 1 Master's degree - UFRJ; Doctoral degree - USP; Associate Professor - UERJ and UNIRIO; Adjunct Professor, Rio de Janeiro State University; 2 Associate Professor, Rio Grande do Sul Federal University. Professor colaborador, Rio de Janeiro State University

**Keywords:** oroantral fistula, surgical flaps, sinusitis

## Abstract

The oroantral fistula is a pathological connection between the maxillary sinus and with the oral cavity. The condition mostly follows dental extraction.

**Aim:**

To present the experience of 25 cases.

**Material and methods:**

Retrospective cases between 1996–2000. The ORL examination included nasal or sinusal endoscopy, a CT scan and histopathological analysis.

**Results:**

Twenty-five cases were found: ten 2nd molar cases, eight 1st molar cases, six 2nd premolar cases, and one canine case. All patients underwent a Caldwell-Luc operation plus excision of the epithelium lining the fistula, that was then completely covered by a flap of mucosa rotated from the genian region.

**Discussion:**

In cases of major fistulae a bone autograft taken from the anterior sinus wall was used. Bacterial cultures (n=19) revealed streptococus pneumoniae (13), haemophillus influenza (6), Moraxella catharralis (2) and staphylococus aureus (2). Aspergillus niger was found in one case presenting as a “fungic ball”.

**Conclusions:**

The only case of surgical failure, after 30 days postoperatively, was reoperated, using a bone graft. After a 6-month follow up all of the patients progressed satisfactorily, including the reoperated patient.

## INTRODUCTION

There are three types of maxillary sinus floor fistulae: the oronasal, the oroantral and the oroantronasal fistula.^1^ The oroantral fistula (OAF) is a pathological communication between the maxillary sinus and the oral cavity,^1^^,^^2^^,^^3^ mostly of the alveolar type, resulting from trauma from endodontic treatment or tooth extraction^1^^,^^4^, ^5^, ^6^, ^7^ that leaves a gap in the bone on the maxillary sinus floor. This gap becomes contaminated by food and saliva, leading to bacterial infection, impaired healing and chronic sinusitis. The OAF occurs mostly in the second and first molars, and the second premolar teeth.^4^^,^^5^ It is more frequent in males compared to females.^1^^,^^8^ Usual radiologic findings in bone include sinus floor discontinuity, a communication between the oral cavity and the sinus, opacification of the sinus, focal alveolar atrophy, and associated periodontal disease.^9^ Antibiotics are of no use for closing the fistulae. Small fistulae tend to heal spontaneously, whereas larger fistulae rarely heal.^8^ Surgery is indicated if a fistula does not heal within three weeks.^1^^,^^4^^,^^8^

Surgery aims to promote ventilation and aeration of the maxillary sinus, to remove diseased bone and to resect the thickened epithelium along the borders of the fistula. Surgical success depends on the technique, the size and site of the fistula and the presence or absence of sinus disease.^4^^,^^8^ It is rare for the sinus to be infection-free in this condition. Sinus disease is commonly treated surgically by a maxillary sinusectomy, according to the Caldwell-Luc technique, followed by middle meatotomy.

Fistula may be closed by using alveolar, palatal or jugal mucosa flaps, after having removed the diseased mucosa and bone.^1^^,^^5^

No surgical flap is superior to another;^2^^,^^4^ each one has its advantages and disadvantages. The palatal flap has better blood perfusion, but the technique is difficult and time-consuming. These flaps are preferred and indicated for wide, high output fistulae. In such cases, the palatal bone structure is exposed, leading to an increased healing time, meaning that the postoperative period is prolonged and troublesome.^6^ The gingivolabial sulcus is narrowed in the oral mucosa flap, which might lead to a second procedure or definitive sequelae.^1^, ^2^, ^3^^,^^5^ An alveolar flap may be used for the treatment of small fistulae.^2^ The jugal mucosa flap is well irrigated and has a higher possibility of covering the fistula and the bone graft.^8^ Special attention must be given to avoid injuring Stenon's duct. The disadvantage of this flap is that it crosses and partially obliterates the gingivolabial sulcus, making it difficult to use prostheses; the flap is also under stress from continuous lip and cheek movements. Furthermore, this operation requires a second procedure to release the sulcus. Palatal rotation may be indicated if the buccal flap is unsuccessful.^3^^,^^7^ This may be problematic in the presence of a third molar fistula, as flap rotation may affect negatively the vascular pedicle.^6^ This flap may result in arterial injury and hemorrhage; it is also more technically challenging and may expose more of the palatal bone if the fistula is larger, requiring a longer procedure and a second operation.^10^

A variety of graft materials have been used in place of bone, including tantalum and gold plates, fascia lata, dura mater and freeze-dried collagen.^1^ In general, allografts are preferred to heterografts.^4^ Although much used in facial bone reconstruction, albeit costly, porous block hydroxyapatite does not yield good results when in contact with the oral mucosa, and is not recommended for the treatment of fistulae.^11^ An original technique is that which transplants a closed apex third molar to the dental extraction site to occlude the gap and close the fistula.^12^

Given the lack of standard protocols for choosing the best technique, the authors present the results of the genian mucosal flap rotation surgery for the treatment of alveolar type oroantral fistulae.

## SERIES AND METHOD

**Figure 1:**
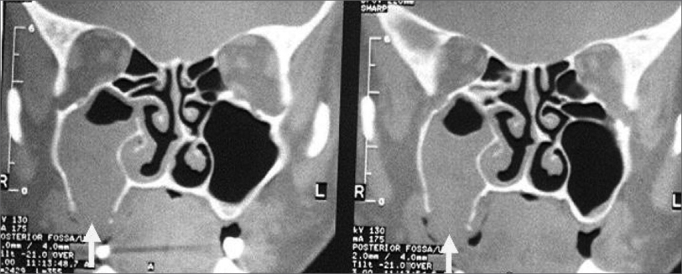
Opacification and erosion of the lower maxillary sinus bone wall.

A retrospective study was made based on compiled data from the charts and medical files of patients diagnosed with alveolar type OAF and treated surgically between 1996 and 2000 at the University Hospital Otorhinolaryngology Unit and at a private clinic. The routine diagnostic sequence included an otorhinolaryngological exam, rigid telescope (30°/4mm) nasal endoscopy, and computed tomography showing coronal, axial and in some cases sagittal sections of the paranasal cavities (Figure 1). Sinus telescope (30°/2.7mm) endoscopy was done if fistulae were equal to or larger than 0.5 mm. Additionally, resected tissue was tested for bacteria and fungi, and sent for pathology.

**Figure 2:**
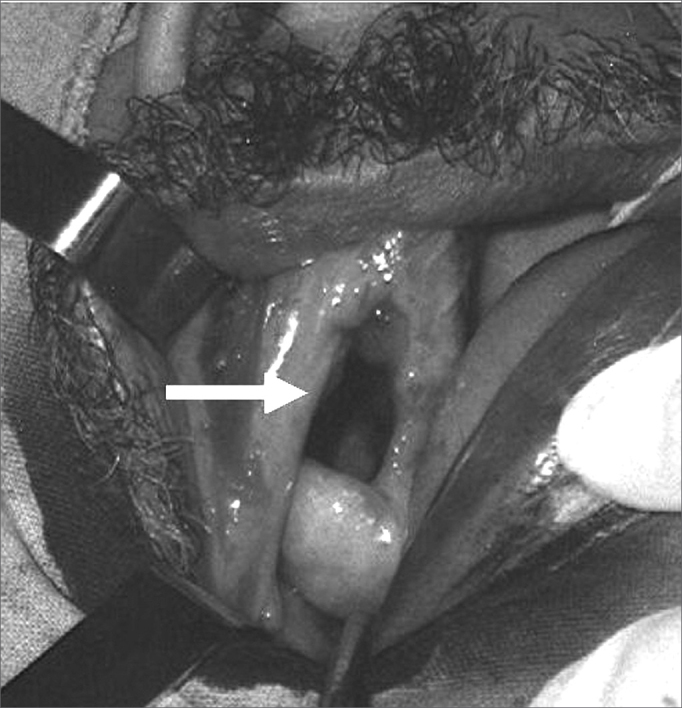
Ample fistula measuring over 1 cm following tooth extraction.

A standard procedure for treating alveolar type OAF at our unit was used in all cases. Patients were operated under general anesthesia and orotracheal intubation by the Caldwell-Luc type maxillary sinusectomy technique. This technique includes stripping down the bone margins and the mucosa of the fistula to healthy tissue, followed by intranasal middle meatotomy and rotation of a contiguous genian mucosal flap of variable width and adequate blood supply. In high-output fistulae (equal to or > 0.5 cm) (Figure 2), a bone graft removed from the anterior wall of the maxillary sinus was placed on the site. Postoperative care included antibiotics during 14 days, instructions to avoid tooth brushing or touching the site with the tongue, to avoid blowing and exercising the cheek or using dental prostheses for seven days. Patients were seen postoperatively on the 7th, 14th, 30th and 180th day. After 30 days a second surgical procedure was undertaken under local anesthesia to release the pedicle of the flap that was linked to the jugal region, followed by hemostasis and suturing.

## RESULTS

There were 25 cases of alveolar fistulae, as follows: 10 second molar fistulae, 8 first molar fistulae, 6 second premolar fistulae and 1 canine fistula. There were 14 cases of high-output secretion fistulae equal to or larger than 0.5 cm. In 23 cases, patients reported previous attempts to correct the fistula, such as allowing second intention healing (10 cases), suturing with simple interrupted stitches to bring together the edges of the fistula (8 cases) and cauterization with a variety of chemical substances (5 cases). Only two cases were referred to an otorhinolaryngologist as the first therapeutic measure. The time needed for diagnosis varied from 21 to 137 days. Three cases had already undergone surgery at another institution.

**Figure 3:**
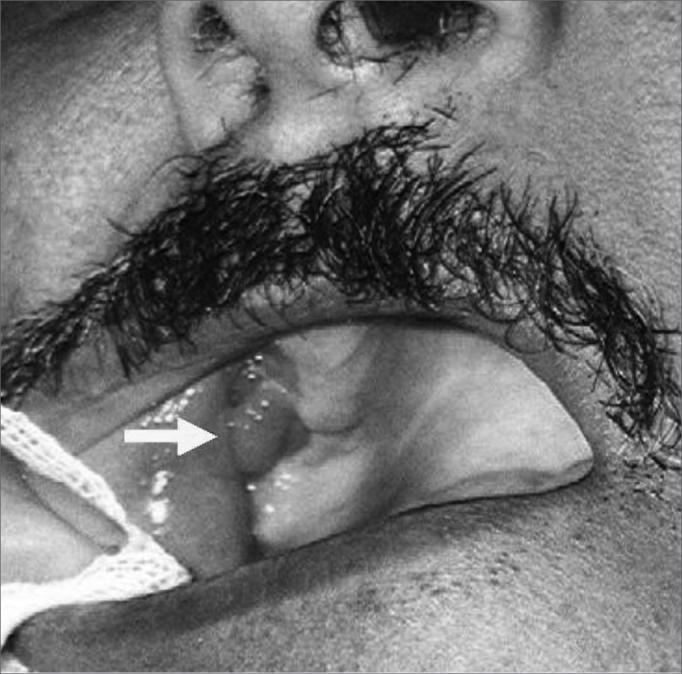
Final aspect of the flap after 30 days.

All of the cases were operated by the Caldwell-Luc type maxillary sinusectomy technique, including stripping of mucosal and bony edges of the fistula to healthy tissue, intranasal middle meatotomy and rotation of a genian mucous flap measuring 1 to 3 cm in width (Figure 3). A bone graft was place on the site in high-output fistula or those that measured 0.5 cm or more (14 cases); bone was removed for grafting from the anterior maxillary sinus wall. All of the cases progressed favorably, except for one. A good result was considered as clinically verified absence of a fistula and of sinusitis.

**Figure 4:**
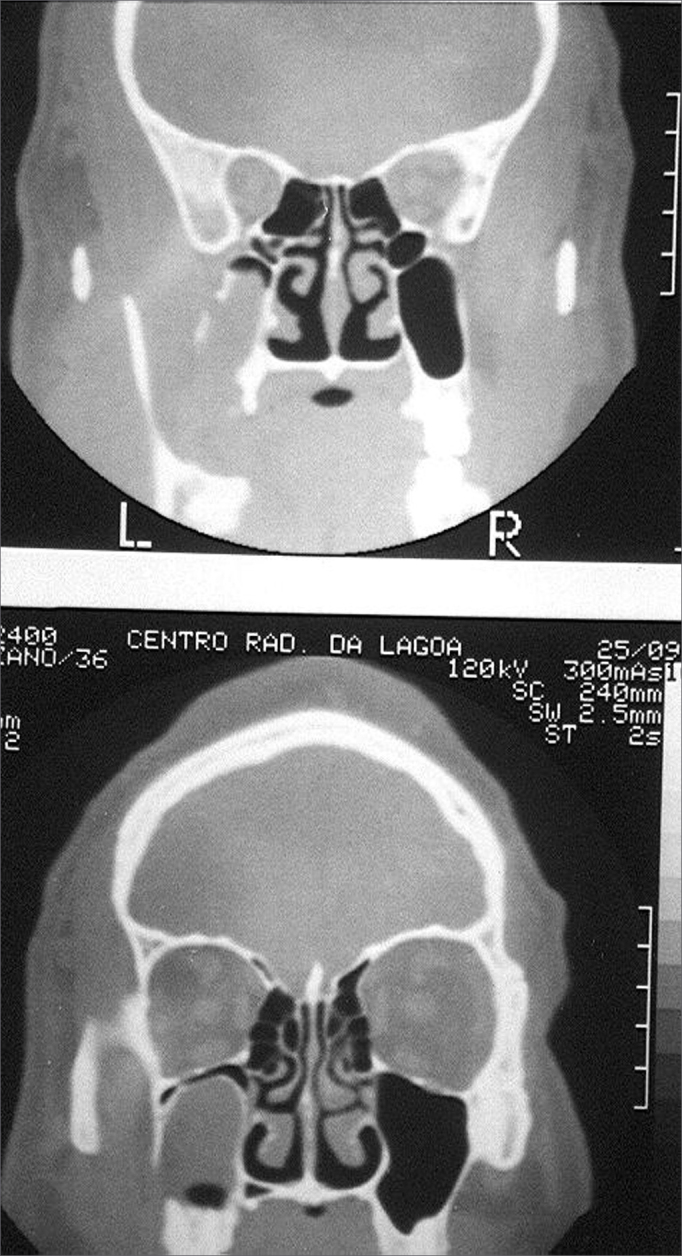
Fistula and an image of a mycetoma in the sinus.

Bacteriological cultures were done in 19 cases that presented secretion with pus, revealing Streptococcus pneumoniae (13 cases), Haemophilus influenzae (6 cases), Moraxella catarrhalis (2 cases), and Staphylococcus aureus (2 cases). Anaerobes were not investigated. Investigation for fungi was done in one case that had a radiologic image of a mycetoma and a suspect dark green tissue within the sinus; this was found to be Aspergilus niger (Figure 4). Pathology of 25 cases revealed non-specific chronic inflammation of variable degree.

A second surgical procedure was done in 19 patients. Six patients chose not to undergo the second procedure, considering themselves pleased with the first operation.

Results were good in all cases 30 days after treatment, except for one case. This patient was reoperated using the same technique, this time with a bone graft, which was successful. After 60 days this patient had progressed favorably. Six months following the treatment, 23 patients were examined and considered as cured. Two patients could not be contacted by telephone or telegrams.

## DISCUSSION

It seems that there is a delay in the diagnosis of oroantral fistulae caused by manipulation of teeth; frequently dentists chose inefficient treatments, such as chemical cauterization, second intention healing and simple interrupted sutures. No study has been conducted on how many of these patients achieved success with the abovementioned methods; such a study would require a comprehensive approach to dental offices and outpatient clinics. More than 30 days with no resolution of the fistula seems to us an excessive time period, particularly considering that these patients end up developing maxillary sinus infection originating from direct contamination from food, and thus require both medical and surgical treatment.

Antibiotics can resolve sinusitis, but have no influence on fistula closure.^8^ Small fistulae tend to heal spontaneously, as opposed to fistulae measuring over 5 mm.^8^ Von Wowern^13^ investigated 90 cases and concluded that spontaneous closure of an OAF of any size was rare, and that surgery was required for closure. Abuabara et al.^14^ found that fistula smaller than 2 mm tended to close, contrary to fistula larger than 3 mm. Sinus disease or foreign bodies impair closure. We found no sinusal foreign bodies. Surgical treatment is usually indicated after three weeks from the diagnosis.^1^^,^^4^^,^^8^ All of the patients had been treated previously by dental surgeons, and as we have no experience with their approaches, we could not evaluate whether the foregoing methods resulted in closure or not; we dealt only with those cases that were referred to us. As surgical treatment involves accessing the maxillary sinus, we believe that otorhinolaryngologists should undertake this procedure, since these professionals are able to deal with sequelae or complications of sinusectomies.

The surgical principles of this operation seek to attain solid and final closure of the fistula and to cure sinus disease. Success depends on the technique, the size and the location of the fistula, as well as the presence or not of sinus disease.^4^^,^^8^

Although rare, it is worth remembering a case that was described in the literature in which the patient developed a subdural empyema, left hemiplegia, and eventual death as complications of a fistula.^15^ We believe that this is another reason for referring the surgical treatment of these cases to physicians.

One of our cases presented a mycetoma, seen on radiology (Figure 3); it appears that the incidence of sinusal aspergillosis is increasing, usually resulting from inhalatory contamination, which has been observed even in non-immunodeficient patients.^16^ A fistula communicating the oral cavity to the maxillary sinus creates an access route for fungal penetration into the sinus, although the origin of the infection could not be established in the case above. Surgical treatment is mandatory and solves the problem; systemic antifungal medication is used only in specific cases, if needed. These are usually severe cases of this disease in immunodeficient patients.^17^ In our patient, abundant rinsing of the sinus with saline and topical antifungal medication solved the problem, and flap rotation closed the fistula successfully.

Although there was one canine OAF case that resulted from traumatic tooth extraction, fistulae of the canine tooth are infrequent. We found cases reported in veterinary journals, which describe this condition as relatively common in small carnivorous animals following tooth extraction. It is interesting to note that repair techniques are similar to those we use.^18^ Normally, the canine relation with the maxillary sinus is not maintained; our one case was an anatomical variant. We opted to place a bone graft in this case, for safety, although it was a low-output fistula.

Many techniques, including flaps, have been proposed for the closure of OAFs.^8^

Our unsuccessful case was a small fistula; later on the patient, who was a nurse, told us that she cleaned the site many time a day with cotton swabs imbibed in oxygenated water. Possibly constant trauma on the loose flap might have increased the diameter of the fistula.

It is rare not to have sinusitis when there is a fistula. Therefore, it is essential to establish the extension of sinus disease, which is usually restricted to the maxillary sinus. Sinusitis was present in all of our cases, which is why we chose the Caldwell-Luc technique for providing the best access to the floor of the sinus for complete cleaning.

The choice of the procedure is controversial. Some prefer simple interrupted sutures (60%), followed by a variety of flaps (39%)^14^. Most of the authors prefer flaps, with no preference for any specific type.^2^^,^^4^ All of them have their advantages and disadvantages.^1^^,^^2^^,^^3^^,^^5^ Güven^8^ treated 90 patients (out of a 98-case series) by Rehrmann's technique, using an advancement buccal flap similar to the one we used. In his series, complications were loss of sensitivity of the infraorbital nerve (2 cases), a reduced gingivolabial sulcus (6 cases), the need for resuturing the flap (3 cases) and granulation tissue along the suture (2 cases).

Similarly, we chose the jugal mucosa flap for all of the cases; this flap is wider, well irrigated and offers a better chance for covering the whole fistula, including the bone graft. The flap should be rotated and fixed free from any stretching force. All of the diseased bone should be removed, including any bone spiculae that might exert pressure on the flap. Special efforts were made to avoid injuring Stenon's duct, so that we did not have this complication. The disadvantage of this flap is that it crosses and partially obliterates the gingivolabial sulcus; thus, prostheses are difficult to use and the flap is under stress from continuous lip and cheek movements. It also requires a second procedure to release the sulcus.

There are various other techniques that might be used in such cases, such as three-layer closure^19^ and third-molar transplantation for closure of the fistula^12^; both techniques have been investigated in small series.

In general, any communication between the maxillary sinus and the oral cavity lasting for more than three weeks should be surgically repaired. It is also essential to cure the sinus disease; otherwise closure of the fistula will not take place.

## CONCLUSION

Our results with a simple rotation of a jugal mucosal flap were good in 96% of 25 cases during the first 30 days, and good in 100% after 60 days, although a larger number of cases would be necessary to critically assess the efficacy of this technique.

## References

[1] Yilmaz T, Suslu AE, Gursel B (2003). Treatment of oroantral fistula: experience with 27 cases. Am J Otolaryngol.

[2] Bluestone CD (1971). The management of oroantral fistulas. Otolaryngol Clin North Am.

[3] Amaratunga NA (1986). Oro-antral fistulae. A study of clinical, radiological and treatment aspects. Br. J Oral Maxillofac Surg.

[4] Haas R, Watzak G, Baron M, Tepper G, Mailath G, Watzek G (2003). A preliminary study of monocortical bone grafts for oroantral fistula closure. Oral Surg Oral Med Oral Pathol Oral Radiol Endod.

[5] Killey HC (1972). Kay LW Observations based on surgical closure of 362 oroantral fistulas. Int Surg.

[6] Lee JJ, Kok SH, Chang HH, Yang PJ, Hahn LJ, Kuo YS (2002). Repair of oroantral communications in the third molar region by random palatal flap. Int J Oral Maxillofac Surg.

[7] Anavi Y, Gal G, Silfen R, Calderon S (2003). Palatal rotation-advancement flap for delayed repair of oroantral fistula. A retrospective evaluation of 63 cases. Oral Med Oral Pathol Oral Radiol Endod.

[8] Güven O (1998). A clinical study on oroantral fistulae. J Craniomaxillofac Surg.

[9] Abraham JJ, Berger SB (1995). Oral-maxillary sinus fistula (oroantral fistula): clinical features and findings on multiplanar CT. Am J Roentgenol.

[10] Car M, Juretic M (1998). Treatment of oroantral communications after tooth extraction. Is drainage into the nose necessary or not? Acta Otolaryngol.

[11] Cottrell DA, Wolford LM (1998). Long-term evaluation of the use of coralline hydroxyapatite in orthognathic surgery. J Oral Maxillofac Surg.

[12] Kitagawa Y, Sano K, Nakamura M, Ogasawara T (2003). Use of third molar transplantation for closure of the oroantral communication after tooth extraction: a report of 2 cases. Oral Surg Oral Med Oral Pathol Oral Radiol Endod.

[13] Von Wovern (1982). Closure of oroantral fistula with buccal flap: Rehrmann versus Môczár. Int J Oral Surg.

[14] Abuabara A, Cortez ALV, Passeri LA, de Moraes M, Moreira RWF (2006). Evaluation of different treatments for oroantral communications: experience of 112 cases. Int J Oral Maxillofac Surg.

[15] Woolley EJ, Patel M (1997). Subdural empyema resulting from displacement of a root into the maxillary antrum. Br Dent J.

[16] Shams MG, Motamedi MH (2003). Aspergilloma of the maxillary sinus complicating an oroantral fistula. Oral Surg Oral Med Oral Pathol Oral Radiol Endod.

[17] Schulte B, Beyer D (1992). Radiologic diagnosis of maxillary sinus aspergillosis. Radiologe.

[18] Smith MM (2000). Oronasal Fistula repair. Clin Tech Small Anim Pract.

[19] Pandolfi PJ, Yavuzer R, Jackson IT (2000). Three-layer closure of an oroantral-cutaneous defect. Int J Oral Maxillofac Surg.

